# Supplementary material for: HIVprotI: an integrated web based platform for prediction and design of HIV proteins inhibitors
Source: J Cheminform. 2018 Mar 9;10:12. doi: 10.1186/s13321-018-0266-y (PMC5845081; doi:10.1186/s13321-018-0266-y)
Supplement: Supplementary file 2 — Additional file 2. Source code of HIVProtI web server. [file 13321_2018_266_MOESM2_ESM.zip › HIVprotI_Source-code/hivprotI/weka-3-6-6/doc/weka/gui/graphvisualizer/LayoutCompleteEventListener.html]

LayoutCompleteEventListener


---


|  |  |  |
| --- | --- | --- |
| **Overview** \| **Package** \| **Class** \| **Tree** \| **Deprecated** \| **Index** \| **Help** \| **Weka's home** | |  |
| **PREV CLASS**   **NEXT CLASS** | **FRAMES**    **NO FRAMES**     **All Classes** |  |
| SUMMARY: NESTED \| FIELD \| CONSTR \| METHOD | DETAIL: FIELD \| CONSTR \| METHOD |  |


---


## weka.gui.graphvisualizer Interface LayoutCompleteEventListener

**All Known Implementing Classes:**: GraphVisualizer, GUI

---

``` public interface LayoutCompleteEventListener ```

This interface should be implemented by any class
which needs to receive LayoutCompleteEvents from
the LayoutEngine. Typically this would be implemented
by the Visualization class.

**Version:**
:   $Revision: 1.5 $ - 24 Apr 2003 - Initial version (Ashraf M. Kibriya)

**Author:**
:   Ashraf M. Kibriya (amk14@cs.waikato.ac.nz)

---

| **Method Summary** | |
| --- | --- |
| `void` | `layoutCompleted(LayoutCompleteEvent le)` |

**Method Detail**

### layoutCompleted

```
void layoutCompleted(LayoutCompleteEvent le)
```


---


|  |  |  |
| --- | --- | --- |
| **Overview** \| **Package** \| **Class** \| **Tree** \| **Deprecated** \| **Index** \| **Help** \| **Weka's home** | |  |
| **PREV CLASS**   **NEXT CLASS** | **FRAMES**    **NO FRAMES**     **All Classes** |  |
| SUMMARY: NESTED \| FIELD \| CONSTR \| METHOD | DETAIL: FIELD \| CONSTR \| METHOD |  |


---
